# Preying dangerously: black widow spider venom resistance in sympatric lizards

**DOI:** 10.1098/rsos.221012

**Published:** 2022-10-19

**Authors:** Vicki L. Thill, Haley A. Moniz, Mike B. Teglas, McKenzie J. Wasley, Chris R. Feldman

**Affiliations:** ^1^ Department of Biology, University of Nevada, Reno, NV 89557, USA; ^2^ Program in Ecology, Evolution and Conservation Biology, University of Nevada, Reno, NV 89557, USA; ^3^ Department of Agriculture, Veterinary and Rangeland Sciences, University of Nevada, Reno, NV 89557, USA; ^4^ United States Fish and Wildlife Service, Klamath Falls Fish and Wildlife Office, Klamath Falls, OR 97602, USA

**Keywords:** adaptation, chemical ecology, muscle histology, predator–prey relationship, *α*-Latrotoxin

## Abstract

Lizards and spiders are natural adversaries, yet little is known of adaptations that lizards might possess for dealing with the venomous defences of spider prey. In the Western USA, two lizard species (*Elgaria multicarinata* and *Sceloporus occidentalis*) are sympatric with and predate western black widow spiders (*Latrodectus hesperus*). The consequences of black widow spider venom (BWSV) can be severe, and are well understood for mammals but unknown for reptiles. We evaluated potential resistance to BWSV in the lizards that consume black widows, and a potentially susceptible species (*Uta stansburiana*) known as prey of widows. We investigated BWSV effects on whole-animal performance (sprint) and muscle tissue at two venom doses compared with control injections. Sprint speed was not significantly decreased in *E. multicarinata* or *S. occidentalis* in any treatment, while *U. stansburiana* suffered significant performance reductions in response to BWSV. Furthermore, *E. multicarinata* showed minimal tissue damage and immune response, while *S. occidentalis* and *U. stansburiana* exhibited increased muscle damage and immune system infiltration in response to BWSV. Our data suggest predator–prey relationships between lizards and spiders are complex, possibly leading to physiological and molecular adaptations that allow some lizards to tolerate or overcome the dangerous defences of their arachnid prey.

## 1. Introduction

Antagonistic relationships, such as those between predator and prey, can have life and death outcomes, thereby exerting intense selective pressures on the species involved [1–3]. In many predator–prey systems, ecological interactions are chemically mediated [4,5] requiring one or both natural enemies to avoid or mitigate the toxins (i.e. poison or venom) they face from their ecological partner [1,4,6]. Given the right ecological and evolutionary conditions, physiological resistance towards toxins may then evolve, as seen across diverse predator–prey systems [6]. Examples include pit viper venom resistance in squirrel [7,8] and opossum prey [9,10], scorpion venom resistance in grasshopper mice predators [11,12], resistance to toad poisons in predatory snakes and lizards [13,14] and resistance to newt neurotoxins in garter snake predators [15,16]. Despite these remarkable examples, we still know little about adaptive toxin resistance in most predator–prey systems. Lizards and spiders represent natural adversaries that have been long overlooked. Here, we test the notion that ecological interactions between venomous spider prey and their lizard predators have led to the evolution of adaptive venom resistance in lizards.

Lizards are a diverse and widespread group of reptiles that are important consumers of arthropods [17–20]. In fact, lizards appear to be particularly important predators of arachnids [21,22], regulating the abundance, richness and diversity of spiders in certain communities [20,23–26]. However, this relationship is not unidirectional, as most spiders are armed with venom [27] and some are major predators of small vertebrates [28–30] including lizards [31,32]. Surprisingly, little work has focused on adaptations that might facilitate the predator–prey relationship between lizards and spiders, which is probably chemically mediated (via spider venom). Thus, it remains unknown whether lizards have evolved specialized adaptations to tolerate or overcome the venom of their spider prey. We describe a previously unexplored system involving potential spider venom resistance in sympatric lizard predators.

Southern alligator lizards (*Elgaria multicarinata*) are known to consume dangerous western black widow spiders (*Latrodectus hesperus*) [33–35] and even seek out their toxic egg sacs [33,36]. Similarly, diet studies on the western fence lizard (*Sceloporus occidentalis*) suggest they regularly consume spiders [23,37], and we have observed *S. occidentalis* readily take *L. hesperus* in captivity (CRF and VLT 2015, 2017, personal observation). In addition, *L. hesperus* tends to be locally abundant and occupies the same microsites as both lizard species (e.g. in the openings of small burrows, under stones, inside log hollows etc.) [38–40]. Even if predation events involving *L. hesperus* are rare, the potency of black widow spider venom (BWSV) may be an important selective pressure on some predatory species, as is the case with kingsnakes (*Lampropeltis*) that infrequently take venomous rattlesnake (*Crotalus*) prey [41]. In fact, small vertebrates appear to be infrequent but important dietary components of widow spiders [42,43], ranging from lizards and snakes to mammals [29,44–48]. Indeed, young *E. multicarinata* have been caught and consumed by *L. hesperus* [47], demonstrating the complex and potentially reciprocal relationship between prey and predator in this system.

The potency of BWSV is high, with an intraperitoneal mouse LD_50_ (lethal dose for 50% of individuals) of 0.64 mg kg^−1^ [49]. By comparison, it takes slightly more venom (LD_50_ of 0.72 mg kg^−1^) from the western diamondback rattlesnake (*Crotalus atrox*) to achieve the same degree of lethality [50]. *Latrodectus hesperus* is also capable of venom metering, with an average venom delivery of 0.016 mg and a known maximum of 0.142 mg in a single bite [51]. If lizards are as susceptible as mammals, this maximum amount of BWSV should be enough to kill up to seven adult *E. multicarinata* (based on a mean adult size of 30 g from our sample) and over 10 adult *S. occidentalis* (based on a mean adult size of 13 g from our sample).

Beyond potency, the venom of *L. hesperus* contains three taxon-specific sets of protein elements that target each of the major prey groups: latroinsectotoxins, affecting insects; latrocrustatoxins, affecting crustaceans; and *α*-Latrotoxin (LTX), affecting vertebrates [43,52–54]. The vertebrate-specific component, LTX, operates by forming cation channels in presynaptic membranes of the neuromuscular junction, forcing massive neurotransmitter release and simultaneously blocking the action of neuromediators [35,52,53]. This neurotransmitter release translates to clinical effects characterized most often by severe muscle cramping [55], muscle fasciculation, local paralysis and pain lasting for hours to days [56,57]. The venom also causes muscle necrosis and infiltration by immune system cells (i.e. eosinophils) [58].

For a small lizard, tackling this relatively large and chemically defended meal may be risky. Black widow spiders are capable of delivering defensive bites during lizard predation (at least under captive conditions) (CRF and VLT 2015, 2017, personal observation). At worst, envenomation could result in death, and even non-lethal bites might injure or temporarily immobilize a lizard, rendering it vulnerable to predation or harsh environmental conditions. If *E. multicarinata* and *S. occidentalis* engage regularly with dangerous prey such as black widows, they may have evolved tolerance or even countermeasures that reduce or negate the effects of envenomation (note that ingested venom is harmless [59]). To determine whether lizard species have evolved mechanisms to overcome BWSV, we exposed three insectivorous lizard species that are sympatric with *L. hesperus* to standardized doses of BWSV and assayed whole-animal sprint performance. We then used comparative histology on muscle tissue at injection sites to investigate tissue damage and cellular immune response to BWSV. We hypothesize that: (i) the two species (*E. multicarinata, S. occidentalis*) that regularly encounter and predate *L. hesperus* would possess resistance, or at least some degree of tolerance, to BWSV; and (ii) a smaller lizard species (*Uta stansburiana*) known to be prey of *L. hesperus* [48] would be susceptible to BWSV. If lizards are tolerant or resistant to BWSV, we expect no significant reduction in post-injection velocity and little to no evidence of muscle tissue damage or increased immune activity compared with controls. By contrast, we expect substantial reductions in post-injection velocity and significant tissue damage and immune response in susceptible lizards. To our knowledge, this is the first study to quantify resistance to any spider venom in natural lizard predators, and the resulting data will help us understand if sympatric lizards have evolved specialized abilities (i.e. toxin resistance) to cope with dangerous prey.

## 2. Material and methods

### 2.1. Animal collection and care

**Figure 1.**
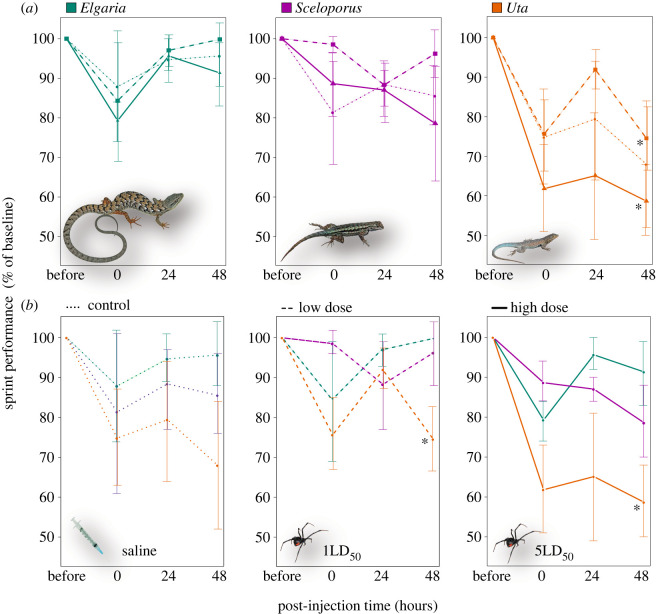
Whole-animal performance (sprint) in response to control (saline injection), low (1LD_50_) or high (5LD_50_) treatments of venom from the western black widow spider (*Latrodectus hesperus*). Changes in sprint speed quantified as the difference in post-injection velocity compared with baseline sprint speed (pre-injection), and recorded across three time points (0: immediately after injection; 24: 1 day after injection; 48: 2 days after injection). (*a*) Plots grouped by species: *Elgaria multicarinata* (southern alligator lizard); *Sceloporus occidentalis* (western fence lizard); *Uta stansburiana* (side-blotched lizard). (*b*) Plots grouped by treatment. Note that *U. stansburiana* is the only species that showed significant reduction in sprint speed compared with baseline speed (table 1), denoted by * (*p* < 0.05). Photos courtesy RW Hansen.

We collected 47 lizards (16 *Elgaria*, 16 *Sceloporus* and 15 *Uta*) (figure 1) from field sites in California and Nevada (electronic supplementary material, table S1) and transported animals to the University of Nevada Reno (UNR). We housed lizards individually in 5- or 10-gallon glass tanks with 40 watt heat bulbs and UV light (Reptisun, 10.0 UVA/UVB, ExoTerra). We maintained lizards on a 12L : 12D light cycle in a room with a mean temperature of 24°C (± 2°C) and mean humidity of 35% (±5%). Lizard diet consisted of crickets or mealworms with occasional calcium supplementation (Rep-Cal Calcium with Vitamin D, Los Gatos, CA, USA). We recorded snout–vent length to the nearest 0.1 cm monthly and body mass to the nearest 0.05 g every two weeks. All live animal procedures were approved by the UNR Institutional Animal Care and Use Committee (IACUC).

### 2.2. Whole-animal performance

We established baseline velocity performance for each lizard prior to injection of BWSV or saline, and then evaluated changes in velocity performance following injections. We adapted our whole-animal performance assay from the well-developed bioassay used to evaluate tetrodotoxin resistance in garter snakes (*Thamnophis*), under the assumption that highly resistant animals will maintain baseline performance capability when exposed to a standardized dose of toxin, while susceptible animals will display dramatic reductions in performance given a dose of equal potency [15,60].

To measure baseline (pre-injection) and post-injection velocity, we sprinted lizards on a 2.2 m racetrack constructed of high-density polyethylene plastic and removable polyester carpet lining. We recorded lizard body temperatures *in situ* using an infrared heat gun (Etekcity, Anaheim, CA, USA) immediately prior to each trial. Trials were recorded top-down using a HERO4 GoPro (GoPro Inc., San Mateo, CA, USA) at 1060 linear video, 60 frames per second, and each video was analysed for velocity using Physlets Tracker software v. 5.1.1 [61]. We measured distance travelled for every two frames, and the Physlets software calculated velocity using our calibrated distance and video frame rate. Baseline velocity was the average of the top 10 velocity values after outlier removal. Once baseline sprint performance was established, we divided lizards into treatment groups: a low-dose group—1 mouse LD_50_, (0.00064 mg g^−1^); a high-dose group—5 mouse LD_50_ (0.0032 mg g^−1^); a control group that received sterile saline. All low-dose amounts were within the range known to be delivered by *L. hesperus* (0.142 mg or less) [51], while high-dose amounts in heavier lizards exceeded this maximum. We obtained BWSV of *L. hesperus* from SpiderPharm (Yarnell, AZ, USA) as lyophilized 0.5 mg pellets and reconstituted them to a 0.1 mg µl^−1^ stock using sterile saline. We serially diluted this to concentrations appropriate for mass-adjusted doses.

We injected lizards intramuscularly (IM) in the dorsal thigh of the right hind leg using a 3/10 cc disposable insulin syringe with a 31-gauge needle (UltiCare, Excelsior, MN, USA). Though an actual spider bite is likely to occur in the subcutaneous (SubQ) layer between the skin and muscle, our lizards where small and unsedated, making reliable SubQ injection difficult. We therefore used shallow IM injections because these were feasible and consistent, and absorption of the venom into muscle tissue would be similar under both circumstances (i.e. SubQ and IM). We kept injection volumes at or below 0.25% of body weight by volume (as per [62]), and we administered volumes of saline to the lizards in the control group equivalent to the volumes of BWSV that treated lizards received. Following injections, we performed three performance assessments: immediately after injection, 24 h after injection and 48 h after injection. Upon completion of the final performance assessment, we monitored lizards for 4 days before humanely euthanizing and harvesting both hind legs for histological examination. Note that space constraints necessitated two separate trials (in 2017 and 2018) and the results of each were pooled.

To ensure that our venom was acting as intended, we injected four mice (*Mus musculus*) with a 1 mouse LD_50_ dose of BWSV (0.00064 mg g^−1^ of venom). We provided mice with pain medication (Buprenorphin SR-Lab, 1 mg kg^−1^) and monitored them for 24 h before we humanely euthanized them. We used the grimace factor scale [63] to quantify mouse discomfort and ensure that pain medication was working as intended.

### 2.3. Comparative histology

Immediately following euthanasia, we harvested dorsal segments of the *femorotibialis externus* and *iliofemoralis* from both the injected right leg and from complementary area of the uninjected left leg for comparison. However, it should be noted that any possible systemic effects of the venom, as opposed to local effects, might not be captured by comparison of the uninjected contralateral limb (control tissue) with the injected limb (treatment tissue). Formalin-preserved tissues were prepared and stained (haematoxylin and eosin) by IDEXX Laboratories (Sacramento, CA, USA). We compared injected and uninjected muscle tissue for each individual using ImageJ v. 1.52a [64], capturing between three and five images per slide at 100× magnification per limb. We analysed images using a randomized grid system, excluding grids from random selection if more than 10% edge white space was present. We quantified tissue damage using per cent damaged area (PDA; adapted from [65]) and quantified immune system response with nuclear counts (adapted from [66]). We considered muscle tissue damaged if the muscle fibre was clearly undergoing necrosis or if there was evidence of recent regeneration (i.e. centrally located nuclei). We performed nuclear counts and distinguished between ‘normal' nuclei (nuclei found as expected within muscle fibres) and nuclei with an ‘abnormal' morphology. We also included a measure of the ratio of normal to abnormal nuclei (per cent normal nuclei, PNN).

We conducted our measures of PDA and nuclear counts using standard ImageJ [67] and add-on Cell Counter [68]. We averaged PDA and nuclear count variables (normal, abnormal and PNN) across grids within images, with up to five replicates per limb.

### 2.4. Analyses

We analysed all data in R v. 3.6.1 [69]. For whole-animal data, we first conducted linear mixed-effect (LMM) regression models with velocity ratio (post-injection : baseline speed) as the response variable and a variety of fixed effects (treatment, species, time, trial, body condition, sex, temperature, volume of fluid injected as a percentage of body weight). We included interactions between variables and individual as a random effect. To select the best model, we used Akaike's information criterion (AIC; [70]). We then performed additional LMMs at the species level to evaluate finer scale effects. Finally, we used a one-sample *t*-test on each species to determine whether post-injection sprint speeds (at 48 h) differed from baseline speeds for each treatment.

To evaluate significant differences in histological metrics between injected right hind limbs and uninjected left hind limbs, we used *t*-tests grouped by species and treatment. To examine histological differences by species, treatment and interaction between the two, we conducted ANOVAs using only data from injected right legs. We also examined the influence of additional variables (trial, sex, body condition, volume injected, temperature) but none were significant for any ANOVA, and thus dropped.

## 3. Results

### 3.1. Whole-animal performance

All lizards injected with saline or venom behaved normally in their enclosures and did not exhibit obvious ill-effects or discomfort during the monitoring period (i.e. no biting at treated limbs, no visible swelling, no observable difficulties with locomotion and no appetite suppression); all individuals survived treatment. Our four mice were severely impacted, with visible swelling of the injected limb and grimace factor scores of one to two, indicating extreme discomfort [63] despite administration of pain-reducing medications.

The best LMM model to explain variation in our dependent variable of post-injection velocity included species, treatment and time as independent variables with individual as a random effect (electronic supplementary material, table S3). Additional variables were either not significant in any model (trial, sex, body condition, volume injected) or were significant only in poorly performing models (temperature). Temperature, though an important factor in the performance abilities of ectotherms [71], was not a descriptive factor for sprint performance in our trials and was not retained in any top models (electronic supplementary material, table S3). All three focal species had body temperatures in the range of their preferred activity range during performance assessments: *E. multicarinata*, $\bar{x}=28.21^{\circ }C$, s.e. = 0.77 [72]; *S. occidentalis*, $\bar{x}=35.89^{\circ }C$, s.e. = 2.98 [73]; *U. stansburiana,* $\bar{x}=35.84^{\circ }C$, s.e. = 2.94 [74]. Given the highly significant differences in post-injection velocity between species (LMM, $\chi^{2}=54.37$, d.f. = 2, *p* < 0.0001), we conducted linear regressions for each species using variables from the top two LMM models (electronic supplementary material, table S3) and ranked them using AIC (electronic supplementary material, table S2).

Variation in post-injection velocity for *E. multicarinata* ($R_{c}^{2}=0.46$, d.f. = 8) was best described by time (LMM, $\chi^{2}=50.73$, d.f. = 2, *p* = 0.0003) and treatment (LMM, $\chi^{2}=3.71$, d.f. = 2, *p* = 0.15), though treatment did not have a significant effect. The changes in post-injection velocities of *E. multicarinata* were not significantly different across any treatment, and in fact mean sprint speeds slightly increased two days after control injections and low-dose injections (figure 1 and table 1).

**Table 1.** Comparison of mean velocity values (cm s^−1^) for each species, along with post-injection velocities after 48 h (the longest time point following injection) by treatment group (control: saline; low: 1LD_50_; high: 5LD_50_), and per cent change in post-injection velocity. Note *Uta stansburiana* has the highest reduction in velocity following all injections, and is the only species to show a significant decrease in velocity (italics*) from venom treatments (based on one-sample *t*-test).

changes in mean velocity by species and treatment					
species	treatment (*n*)	$\bar{x}$ pre-injection cm s^−1^ (s.e.)	$\bar{x}$ post-injection cm s^−1^ (s.e.)	% change (%)	*p*-value
*Elgaria multicarinata*	control (4)	141.08 (4.41)	157.34 (15.86)	+11.53	0.387
	1LD_50_ (6)	120.9 (6.68)	133.40 (9.17)	+10.34	0.300
	5LD_50_ (6)	142.18 (9.22)	130.20 (7.50)	−8.43	0.092
*Sceloporus occidentalis*	control (4)	158.66 (17.86	135.92 (16.05)	−14.33	0.101
	1LD_50_ (5)	154.21 (17.76)	159.38 (10.90)	+3.35	0.612
	5LD_50_ (5)	163.47 (13.38)	133.84 (22.63)	−18.13	0.097
*Uta stansburiana*	control (4)	168.41 (30.08)	113.91 (23.06)	−32.36	0.065
	1LD_50_ (5)	163.50 (6.53)	121.70 (5.95)	*−25.57*	0.002*
	5LD_50_ (5)	175.32 (14.18)	101.99 (8.11)	*−41.83*	0.003*

Post-injection velocity for *S. occidentalis* ($R_{c}^{2}=0.11$, d.f. = 2) was best described by treatment, which did not have a significant effect (LMM, $\chi^{2}=4.20$, d.f. = 2, *p* = 0.12). Likewise, the average reduction in post-injection velocity was not significantly different across the three treatment groups (table 1). However, compared with *E. multicarinata*, variation in post-injection velocity in *S. occidentalis* was more extensive (figure 1).

*Uta stansburiana* post-injection velocity differences were best described by time and treatment ($R_{c}^{2}=0.73$, d.f. = 8), both significant (LMM,$\;\chi^{2}=107.36$, d.f. = 3, *p* < 0.0001; $\chi^{2}=11.091$, d.f. = 2, *p* = 0.003). *Uta stansburiana* had greater reductions in post-injection velocity than both *E. multicarinata* and *S. occidentalis*, and this pattern extended across all treatment groups (including control). However, only *U. stansburiana* receiving doses of BWSV showed significantly reduced velocities two days post-injection (figure 1 and table 1), with animals subject to 1LD_50_ sprinting roughly 25% slower than baseline (*t* = −7.312, d.f. = 4, *p* = 0.002), and those subject to 5LD_50_ running 42% slower than baseline (*t* = −6.772, d.f. = 4, *p* = 0.003).

### 3.2. Comparative histology

All negative control muscle tissues showed similar histological metrics across species with limited abnormal nuclei counts and PDA as expected in normal muscle tissue. These results indicate that lizards did not experience systemic venom effects, and justify the use of uninjected contralateral limbs as control tissue. Furthermore, muscle tissues that received control injections were not significantly different from uninjected control tissues except in *S. occidentalis*, which had significantly higher abnormal nuclei counts, lower PNN and higher PDA (table 2). The number of normal nuclei were not significantly different between control and injected muscle tissue in any species for any treatment except for *S. occidentalis* in the low-venom group (table 2).

**Table 2.** Comparison of muscle tissue response by leg (L: left leg, un injected; R: right leg, injected), treatment (control: saline; low: 1LD_50_; high: 5LD_50_) and species. Variables include ‘norm', which is a count of normal muscle fibre nuclei; ‘abnorm', which is a count of abnormal nuclei (leucocytes or central nuclei); ‘PNN', or per cent normal nuclei, which is a ratio of normal nuclei to total nuclei, which provides a measure of how normal nuclei are responding and how immune system is responding via abnormal (white blood cell) increase; and ‘PDA', or per cent damaged area, which is a measurement of damaged muscle fibre. Significant differences in italics and denoted by * (*p* < 0.05), ** (*p* < 0.01) and *** (*p* < 0.001) based on ANOVA. Note that significant differences between uninjected (L) and injected (R) tissues were found in *Sceloporus* and *Uta* across many response variables, but only one in *Elgaria*.

muscle tissue response									
species	treatment (*n*)	L $\bar{x}$ norm (s.e.)	R $\bar{x}$ norm (s.e.)	L $\bar{x}$ abnorm (s.e.)	R $\bar{x}$ abnorm (s.e.)	L $\bar{x}$ PNN (s.e.)	R $\bar{x}$ PNN (s.e.)	L $\bar{x}$ PDA (s.e.)	R $\bar{x}$ PDA (s.e.)
*Elgaria multicarinata*	control (25)	54.9 (3.8)	49.1 (2.6)	8.9 (1.2)	19.7 (5.6)	79.87 (2.7)	73.7 (5.1)	0.6 (0.2)	3.3 (1.7)
	low (30)	62.8 (3.5)	8.0 (1.1)	7.6 (1.1)	13.5* (2.6)	88.59 (1.2)	81.0* (1.2)	0.3 (0.1)	2.1 (1.1)
	high (23)	43.9 (3.4)	44.7 (3.7)	10.0 (2.2)	13.0 (2.3)	82.3 (3.4)	77.6 (3.2)	0	0.8 (0.6)
*Sceloporus occidentalis*	control (24)	52.7 (5.0)	50.1 (4.5)	11.4 (2.4)	50.9* (13.6)	84.3 (2.7)	63.7** (5.8)	1.1 (0.5)	11.4* (4.2)
	low (30)	34.1 (1.6)	44.8* (4.1)	9.2 (1.5)	38.0** (9.6)	80.1 (2.7)	67.5** (4.9)	0.2 (0.1)	5.4** (1.8)
	high (30)	49.9 (3.9)	38.9 (4.0)	5.8 (1.2)	61.6*** (9.9)	90.9 (1.4)	48.6*** (6.2)	0.1 (0.1)	24.7** (6.1)
*Uta stansburiana*	control (17)	53.2 (4.7)	45.9 (2.9)	13.0 (3.0)	9.5 (2.0)	83.5 (2.9)	84.1 (2.6)	0.2 (0.2)	0.3 (0.2)
	low (30)	50.8 (2.5)	50.1 (3.6)	9.4 (2.2)	28.6* (7.3)	86.3 (2.3)	72.3** (1.3)	0.2 (0.1)	5.1** (1.8)
	high (28)	58.0 (3.8)	55.5 (4.7)	7.2 (1.1)	62.3** (15.2)	89.8 (1.35)	64.0*** (5.6)	0.4 (0.2)	16.1** (5.0)

Differences in normal nuclei counts appeared to be driven primarily by the interaction between species and treatment (*F*_4,229_ = 2.42, *p* = 0.049), while differences in abnormal nuclei counts were significant among species (*F*_2,229_ = 9.92, *p* = 0.0007) and treatment (*F*_2,233_ = 4.99, *p* < 0.001), with a weak but significant effect from the interaction between species and treatment (*F*_4,229_ = 2.45, *p* = 0.046). Species-level effects were driven mostly by *E. multicarinata*, which had reduced abnormal nuclei compared with both *S. occidentalis* (Tukey HSD, diff = −48.67, *p* < 0.01) and *U. stansburiana* (Tukey HSD, diff = −49.07, *p* = 0.01).

PDA was significantly affected by species (*F*_2,229_ = 9.25, *p* = 0.001), treatment (*F*_2,229_ = 8.29, *p* = 0.003), and the interaction between species and treatment (*F*_4,229_ = 3.20, *p* = 0.01). *Elgaria multicarinata* had significantly lower PDA compared with *S. occidentalis*, especially in high-venom treatments (Tukey HSD, diff = 23.88, *p* < 0.0001). The high-venom treatment group had significantly higher PDA across species compared with low and control groups (Tukey HSD, diff = 8.88, *p* < 0.01).

**Figure 2.**
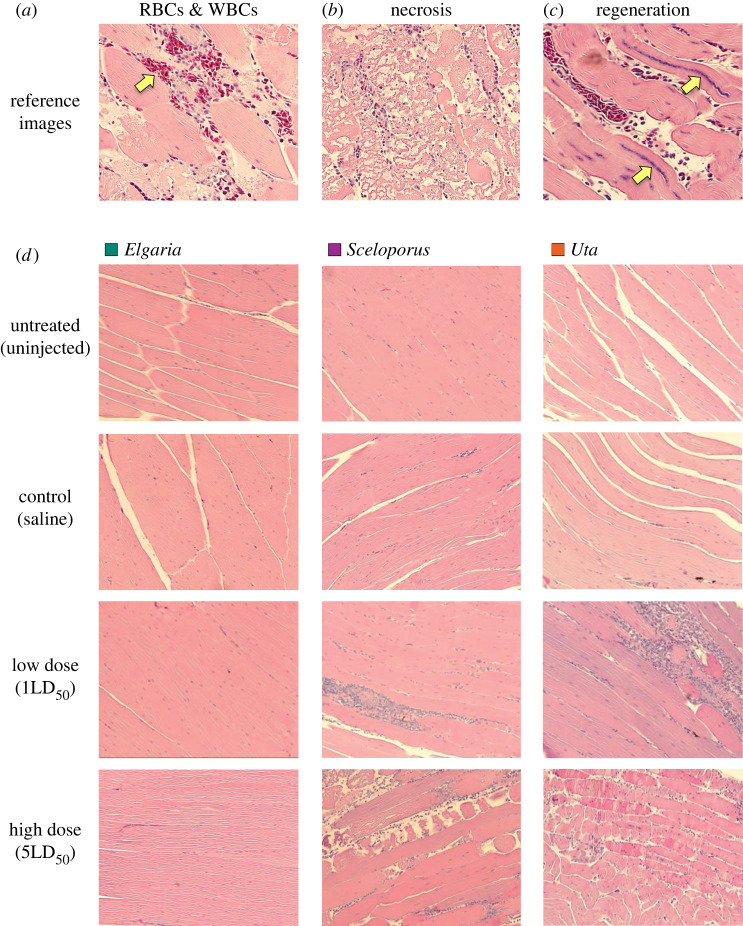
Histological images of lizard muscle tissue taken at 100× magnification. In (*a­*–*c*), images have been cropped to highlight specific morphological characteristics. Arrows point to a raft of nucleated red blood cells with associated white blood cells (*a*) and regenerating muscle fibres (*c*). In (*b*), the focal field is filled with necrotic fibres. Panel (*d*) highlights differences in muscle tissue response by species (columns) and treatment (rows). Note that *E. multicarinata* has tissue with similar appearance for all treatments, while both *S. occidentalis* and *U. stansburiana* show muscle tissue response in the form of necrosis and white blood cell increases in venom treatments.

**Figure 3.**
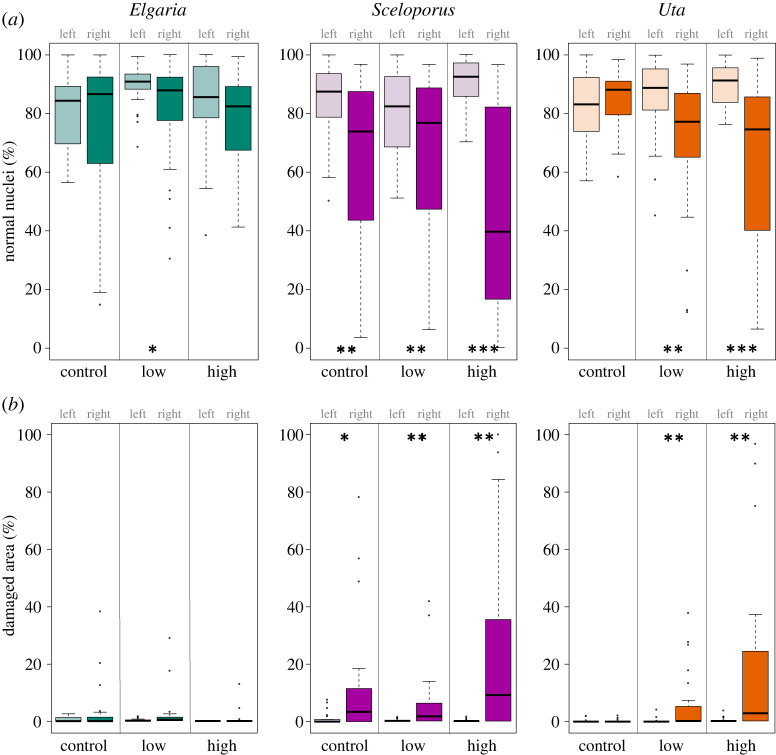
Boxplots of muscle tissue response variables from lizard hindlimbs that were untreated (left leg) and treated (right leg) with either control (saline) low venom dose (1LD_50_) or high dose (5LD_50_). (*a*) Percentage of total nuclei that appear normal (PNN). (*b*) Per cent of damaged area (PDA). Significant differences between untreated (left) and treated (right) tissue denoted by * (*p* < 0.05), ** (*p* < 0.001) or *** (*p* < 0.0001).

We found little evidence that *E. multicarinata* suffered muscle tissue damage or mounted an immune response when comparing untreated and treated tissues (figures 2 and 3, table 2). Specifically, we found no significant difference in right leg muscle tissue between saline controls and venom treatments, with treated limbs averaging between 0.8% and 3.3% PDA across treatments (figure 3 and table 2). While these differences were not significant for any treatment (table 2), *E. multicarinata* did have slightly higher abnormal nuclei counts for low-venom-treated muscle tissue compared with uninjected muscle tissue (*t* = −2.22, d.f. = 59.11, *p* = 0.03; figure 3*b*).

We found evidence that *S. occidentalis* suffers effects from BWSV at the tissue level. Abnormal nuclei counts were significantly elevated in all treated muscle tissue compared with untreated tissue (control: *t* = −3.79, d.f. = 35.78, *p* = 0.0006; low dose: *t* = −3.72, d.f. = 43.43, *p* = 0.0006; high dose: *t* = −7.15, d.f. = 42.47, *p* = 0.0001; figure 3*b* and table 2). This pattern continued for PDA, with all injected muscle tissue showing significant increases compared with untreated muscle tissue; this effect was especially strong in high-treatment muscle tissue (control: *t* = −3.35, d.f. = 34.00, *p* = 0.002; low dose: *t* = −3.74, d.f. = 41.20, *p* = 0.0005; high dose: *t* = −4.98, d.f. = 41.01, *p* < 0.0001; figure 3*d*).

The strongest evidence for tissue-level effects of BWSV was found in *U. stansburiana*. We found significant differences between untreated and venom treated muscle tissue for most muscle tissue response variables. Control tissue had no significant differences compared with untreated tissue except in normal nuclei counts (*t* = 2.60, d.f. = 45.84, *p* = 0.01) (figure 3 and table 2). Abnormal nuclei counts were significantly increased for both venom treatments (low dose: *t* = −2.97, d.f. = 49.77, *p* = 0.005; high dose: *t* = −4.47, d.f. = 40.43, *p* = 0.0006; figure 3*b*). Normal nuclei counts were similar between control and injected muscle tissue. Finally, PDA was significantly increased in venom treatments (low dose: *t* = −3.61, d.f. = 41.63, *p* = 0.0008; high dose: *t* = −4.08, d.f. = 40.10, *p* = 0.0002; figure 3*d*).

## 4. Discussion

Predators that engage venomous prey are expected to evolve mechanisms that help avoid or withstand the effects of envenomation [6,75,76]. Lizards are major predators of spiders [22,25,26], yet almost nothing is known about the abilities of lizards to overcome the venomous defences of spider prey. Here, we used whole-animal assays in conjunction with tissue histology to evaluate potential resistance to BWSV in three lizard species. We found differing abilities to withstand BWSV across all three species, possibly in relation to the nature and intensity of the predator–prey relationship between these lizards and spiders.

### 4.1. Degrees of resistance to black widow spider venom

The southern alligator lizard (*E. multicarinata*) demonstrated a surprising ability to withstand BWSV. This species sprinted just as well after being administered low (1LD_50_) and high (5LD_50_) doses of BWSV as it did during the pre-injection baseline (figure 1 and table 1). Furthermore, histological sections showed no significant difference in tissue damage or immune cell infiltration between untreated (left) and treated (right) muscle tissue or control and treatment muscle tissue (figures 2 and 3, table 2). From a mechanistic perspective, the absence of organ or cellular damage in *E. multicarinata* may provide clues about the molecular or physiological basis of resistance. In similar systems where the mode of venom resistance is at least partially understood, venoms are often prevented from attacking target tissues because they are bound by macromolecules [8,77–79]. These ‘toxin-scavenging' molecules are usually inhibitor proteins that actively circulate in the blood stream (e.g. serum), so that when envenomation occurs, they act immediately to bind and inactivate venoms [6]. Though speculative, perhaps similar mechanisms have evolved in *E. multicarinata* to prevent BWSV from attacking muscle and nerve tissue.

It is interesting to note that *E. multicarinata* is the only one of our lizards documented to consume *L. hesperus* regularly in the wild [33,34]. In fact, *E. multicarinata* was at one point suggested as a potential biological control for *L. hesperus* [33] because of its predatory habits on widow spiders and their egg sacs, and is one of the few lizard species that can be found in the same suburban environments [39,80] (CRF and VLT 2015, 2017, personal observation) that now maintain high densities of *L. hesperus* [33,81]. The ability to consume an abundant but dangerous spider while suffering no ecologically relevant effects of envenomation may be particularly useful in disturbed urban and suburban settings that contain a reduced arthropod prey base [82,83]. Alligator lizards appear to be well-fortified against harmful spider prey. These lizards are protected by osteoderms (bone embedded in the scales) that cover the cranium, dorsum and ventrum [39], probably providing a first line of defence against spider envenomation. If envenomation does occur, *E. multicarinata* seem unfazed by even high doses of BWSV, functioning at five times the mouse LD_50_, roughly equal to six times the average amount delivered by *L. hesperus* (based on mean lizard mass).

While *S. occidentalis* did not show a significant effect of treatment at the whole-animal level (figure 1 and table 1), this species did display significant muscle damage and elevated immune response, especially at the high dose (figure 3 and table 2). Their ability to run at speeds near baseline despite muscle tissue damage suggests these animals might be able to tolerate BWSV, avoiding severe ecological costs of envenomation in natural situations. The physiological effects of BWSV may be localized in *S. occidentalis*, allowing near maximal performance, at least in our brief sprint trials. It is possible, however, that longer trials simulating lengthy predator evasion incidents could reveal performance costs of envenomation. Furthermore, BWSV may still impose metabolic or energetic costs on lizards that must repair and heal injured tissues.

In contrast to *E. multicarinata* and *S. occidentalis*, we expected *U. stansburiana* to be the most affected by BWSV because they are probably too small to prey on widow spiders and given their status as occasional prey to *L. hesperus* [48]. Indeed, *U. stansburiana* showed significantly reduced performance capabilities under BWSV treatments, especially at the highest dose (figure 1 and table 1). These lizards also suffered significantly higher muscle fibre damage and immune system infiltration in treated muscle tissue compared with untreated muscle (figure 3 and table 2). The dramatic reduction in sprint speed in the high-treatment group would almost certainly translate to significant ecological effects, impacting their ability to evade predation, effectively capture prey or perhaps exposing them to unfavourable environmental conditions.

The apparent gradient of resistance to BWSV across species, from very high in *E. multicarinata,* intermediate in *S. occidentalis*, to low in *U. stansburiana*, may relate to the intensity of predator–prey interactions. Perhaps *E. multicarinata* frequently consumes *L. hesperus*, while *S. occidentalis* only occasionally and *U. stansburiana* rarely or never. Likewise, microsympatry might be greatest between *E. multicarinata* and *L. hesperus* [33,39,80], so that young lizards would be vulnerable to widow predation [47] without protection. Assessing these hypotheses will require greater information on the diet and habits of these lizards, as well as their interactions with *L. hesperus*. Furthermore, diet and ecological interactions might vary across the landscape, providing an opportunity to examine spatial variation in predator and prey traits [7,16,84]. On the other hand, our sample of lizards represents two deep clades (Anguiformes: Anguidae: *E. multicarinata*; Iguania: Phrynosomatidae: *S. occidentalis* and *U. stansburiana*) that have been diverging since the Mid-Jurassic [85]. Thus, differences in BWSV resistance across species might simply reflect lineage-specific distinctions in physiology and the detoxification pathways of these two lizard clades.

### 4.2. Potential broad-spectrum venom resistance in lizards

An unexpected outcome of our study was that all lizard species fared well, at least relative to mammalian models. In most mammals, BWSV is potent, causing pain, paralysis and even death [29,56,57]. We administered the equivalent of one or five times the amount of a mammalian lethal dose, yet our lizards displayed no outward evidence of pain, swelling or immobility. All were capable of normal movement, performed multiple sprint trials, and all survived the treatments. These results raise several questions about the generalized ability of lizards to tolerate spider venom. Does a higher tolerance of BWSV in lizards relative to mammals simply reflect underlying differences in the physiologies of reptiles and mammals (e.g. rates at which toxins are metabolized)? Or do lizards possess some baseline or low-grade resistance to neurotoxic arachnid venoms, and perhaps other arthropod toxins, because they have been engaging with spiders and other dangerous arthropods for over 100 Myr [86,87]? Or do these results pertain only to the lizards that are sympatric with *L. hesperus*, suggesting this spider is important as both prey and predator in this system? Further work examining the degree of resistance to various arthropod toxins across a diverse range of reptiles could help us understand the evolution of venom resistance in lizards.

## 5. Conclusion

Predators that interact with chemically defended prey must avoid or mitigate those defences, whether through behavioural changes in prey recognition, handling techniques [88,89], or through biochemical and physiological changes that allow them to reduce or block the effects of toxins [6,75,76,90–92]. Toxin resistance has evolved in many systems, sometimes allowing consumption of prey with neurotoxic secretions [1,13,14,93], neurotoxic venoms [11,12,94–96] or prey that are protected by haemorrhagic venoms [77–79]. Clearly, there are a variety of adaptive pathways that can allow predators or prey to escape the effects of diverse toxins.

Occasionally predator–prey interactions lead to a coevolutionary arms race, where a cyclical escalation in offensive and defensive adaptations continues until some limit is reached or one party somehow ‘escapes' the cycle [3,16,97]. Though there are many predator–prey systems involving chemically defended prey or venomous predators, only rarely have these been shown to be truly coevolutionary [7,16,97]. This work represents a first step in determining whether and how some lizards may have entered a coevolutionary arms race with dangerous arachnid prey.

## Data Availability

We deposited all animals as voucher specimens in the herpetology collection of UNR (electronic supplementary material, table S1) [98]. All data underlying these analyses are available on the Open Science Framework (OSF) digital repository: whole-animal resistance data (https://osf.io/9GJZB/); muscle tissue histology data (https://osf.io/pdg9r/). The data are provided in electronic supplementary material.
